# Multidisciplinary Design–Based Multimodal Virtual Reality Simulation in Nursing Education: Mixed Methods Study

**DOI:** 10.2196/53106

**Published:** 2024-07-26

**Authors:** Ji-Young Yeo, Hyeongil Nam, Jong-Il Park, Soo-Yeon Han

**Affiliations:** 1 College of Nursing Hanyang University Seoul Republic of Korea; 2 Department of Computer Science Hanyang University Seoul Republic of Korea; 3 Department of Nursing Bucheon University Bucheon Republic of Korea

**Keywords:** multidisciplinary, multimodal, nursing, simulation, virtual reality, VR, education, allied health, educational, simulations, pediatric, pediatrics, paediatric, paediatrics, feasibility, nurse, nurses, qualitative, interview, interviews, development, develop, teaching, educator, educators, user test, user testing, module, modules, usability, satisfaction

## Abstract

**Background:**

The COVID-19 pandemic underscored the necessity for innovative educational methods in nursing. Our study takes a unique approach using a multidisciplinary simulation design, which offers a systematic and comprehensive strategy for developing virtual reality (VR) simulations in nursing education.

**Objective:**

The aim of this study is to develop VR simulation content for a pediatric nursing module based on a multidisciplinary simulation design and to evaluate its feasibility for nursing education.

**Methods:**

This study used a 1-group, posttest-only design. VR content for pediatric nursing practice was developed by integrating the technological characteristics of a multimodal VR system with the learning elements of traditional nursing simulation, combining various disciplines, including education, engineering, and nursing. A user test was conducted with 12 nursing graduates (preservice nurses) followed by post hoc surveys (assessing presence, VR systems, VR sickness, and simulation satisfaction) and in-depth, one-on-one interviews.

**Results:**

User tests showed mean scores of 4.01 (SD 1.43) for presence, 4.91 (SD 0.81) for the VR system, 0.64 (SD 0.35) for VR sickness, and 5.00 (SD 1.00) for simulation satisfaction. In-depth interviews revealed that the main strengths of the immersive VR simulation for pediatric pneumonia nursing were effective visualization and direct experience through hands-on manipulation; the drawback was keyword-based voice interaction. To improve VR simulation quality, participants suggested increasing the number of nursing techniques and refining them in more detail.

**Conclusions:**

This VR simulation content for a pediatric nursing practice using a multidisciplinary educational design model was confirmed to have positive educational potential. Further research is needed to confirm the specific learning effects of immersive nursing content based on multidisciplinary design models.

## Introduction

### Overview

Virtual reality simulation (VRS) education, which integrates the latest information and communications technology innovations into simulation education, is a cost-effective solution for nursing practice education. It allows for attainable and predictable results without limitations of time and place. Consequently, VRS is expected to be extensively used in nursing curricula [1]. The demand for immersive education is growing rapidly following the clinical practice challenges experienced during the COVID-19 pandemic [2]. Various studies have been conducted to enhance immersion, a major advantage of virtual reality (VR)–based educational content; increase the educational efficacy of VR-based content; and minimize the side effects of the technology [3].

VR technology for on-site nursing simulation education is in its early stages and is centered on 2D web-based simulations, primarily using computers and monitors [4]. Even in realistic 3D simulation education, most studies are based on fragmentary skill-oriented scenarios using handheld devices [5].

It is essential to clarify the desired direction of immersive education, learning situational elements, and skill levels required for students to use immersive simulations in practical nursing training effectively. This approach should be based on an optimal theoretical framework and educational design [6]. Since immersive simulation is an educational method that integrates elements of traditional nursing simulation education and engineering elements, to maximize the effect of education using immersive simulation, the learning design must consider various elements of each area.

Despite significant advancements in IT and a growing interest in immersive content, little research exists on multidisciplinary design models for immersive education and the feasibility of design model-based content [7-9].

This study aimed to systematically develop 3D nursing simulation content based on a VR educational design model and the National League for Nursing (NLN) Jeffries Simulation Theory. It also conducted user tests to evaluate its feasibility in nursing education. We propose a multidisciplinary VRS content development model and provide the basis for future VR-based nursing education.

### Theoretical Framework

The VR-based nursing simulation content was developed using the NLN Jeffries Simulation Theory [10] and Han’s [11] VR-based Educational Simulation Model. The selection of these 2 theories as a framework for this study was a strategic approach to systematically consider the characteristics of VR media and educational elements to maximize learning effectiveness. Rather than simply borrowing VR technical elements for nursing education, we sought to develop the most effective educational content in a multidisciplinary manner by integrating technology, educational elements, and nursing. The NLN Jeffries Simulation Theory provides a well-established foundation in nursing education, while Han’s [11] VR-based Educational Simulation Model offers specific guidelines for creating immersive and user-centered VR experiences.

The NLN Jeffries Simulation Theory is widely used in nursing education to guide the development of simulation-based learning experiences. This theory presents components as participant factors, facilitator factors, educational strategies factors, and expected outcomes, offering versatility in their application in nursing education. By outlining specific components that can be tailored to the needs and objectives of learners, the theory provides a robust framework for educators. It maximizes the effectiveness of simulation as a teaching strategy, ensuring that educational goals are met and that learners are better prepared for clinical practice. Especially as educational strategies factors, this includes the design and execution aspects of simulation activities, such as the complexity of simulations, feedback mechanisms, and debriefing methods. These factors are key to optimizing the learning environment and supporting the achievement of educational goals. Endorsed by the NLN, this theory has become a cornerstone in the field, guiding educators on how to integrate simulation into their teaching practices effectively.

Han’s [11] research aims to develop and validate design principles for optimizing VR-based educational simulations. It explores ways to use VR to extend users’ learning experiences into realistic contexts. Han’s [11] model comprises 12 design principles based on the 3 categories of contextual scenario, affordance in the simulation, and user activity and response. These categories emphasize creating immersive learning experiences that more closely resemble real-life situations, thereby enhancing the learner’s engagement and facilitating a deeper understanding of the subject matter. The 12 design principles encompass creating realistic scenarios, engaging user actions, and reflective activities closely mirroring real-life contexts. Applying these principles to nursing education simulations is justified as they enhance experiential learning, improve critical thinking and decision-making skills, and provide a safe environment for clinical practice. The principles ensure that the simulations are relevant, technologically apt, and realistically mimic health care settings, thereby making theoretical knowledge tangible and facilitating embodied learning.

This theoretical framework can provide a structured approach to developing simulations that are both educational and reflective of actual nursing practice. Thus, Han’s [11] model has 2 main advantages when applied to develop this nursing simulation content. First, it comprehensively considers educational engineering elements and the technical characteristics of VR. Second, it is subdivided into 3 clear categories and 12 subcategories, specifying the characteristics of each area. This structure allows for the provision of specific and clear guidelines for application in developing this nursing simulation content. We comprehensively considered the characteristics of VR, a technical element, as well as nursing and educational engineering elements, and selected an appropriate model to develop optimal immersive content based on a multidisciplinary perspective (Textbox 1).

**Immersive content development based on virtual reality (VR) education engineering design principles.**

**Principle of replicating real-life problems:**

- To reflect the nature and importance of real-life problems, a contextual scenario was constructed using the clinical pathway of a disease to allow the learner to experience the entire flow of actual clinical practice for a specific disease and the corresponding nursing care.

**Principle of adequacy of VR technology:**

- Technique to perform nursing skills with bare hands without a hand device was applied through a variety of technologies, allowing the learner to have experiences similar to clinical practice.
- Using deep learning technology, the virtual caregiver was configured to give feedback to the learner with a verbal response.

**Principle of similarity to real environment:**

- To construct a practice environment as similar to real-life situations as possible; actual photos were provided as reference materials when creating virtual objects, which was refined through several revisions.

**Principle of structural planning:**

- Based on the Jeffries Simulation Theory template, learning goals for each module were set, upon which simulation elements and specific learning contents were organized.
- A flowchart was designed to show the VR simulation (VRS) content deployment order according to the user’s activities.

**Principle of implementing a professional approach:**

- The content was algorithmically designed to recognize appropriate or inappropriate nursing procedures performed by learners and to provide feedback according to their level of performance, allowing them to ultimately embody the knowledge, skills, attitudes, and so forth, that was required of a nurse.

**Principle of structured activity deployment:**

- Contents and screens were designed according to the expected progress of the learner’s activities, and a storyboard including the main contents was constructed according to the progress of the simulation activities.

**Principle of simple-to-complex process:**

- The modules were structured to align with the sequential order of patient care from admission to discharge, ensuring that activities are carried out in a systematic and sequential manner.

**Principle of virtual recognition:**

- To enhance the learner’s sense of presence and awareness of activity direction in the VR space, the simulation modules were designed to display the learner’s hands or body parts on the screen.

**Principle of the reality of operation and selection:**

- To enable realistic exploration, manipulation, and selection activities, real-life activities and voice responses were presented.
- The nursing outcomes were algorithmically configured to vary depending on the learner’s VRS activities.

**Principle of providing relevant information:**

- The content was designed to assist the learner with the initial orientation and facilitate nursing activities by providing information on the envisaged simulation activities.
- In the introduction stage, instructional materials and preinstructional videos were provided.
- In the activity stage, pop-up quizzes or audio formats were used to provide information on the elements and procedures that the learner needed to consider during simulation performance.

**Principle of promoting critical thinking:**

- The problem situations related to pediatric patients hospitalized with pneumonia, learning objectives, and framework for required nursing interventions were presented.
- Critical thinking was promoted by allowing for decision-making and implementation within the presented situation.
- Use of tablet devices was included in the content to allow the learner to retrieve information on the patient’s conditions and related information whenever necessary.

**Principle of encouraging critical reflection:**

- Feedback was provided to the learner during the simulation process through quizzes or a virtual agent’s responses in a manner to give them the opportunity to reflect on the appropriateness of their own nursing actions.
- A performance checklist was displayed on the screen just before the end of the VRS, and feedback videos were provided after the simulation ended to allow the learner to reflect on their nursing performance again.

### Objectives

Therefore, this study’s theoretical framework, which integrates nursing theory and educational technology models, can provide a structured approach to developing VRSs for educational purposes that reflect actual nursing practice situations.

## Methods

### Study Design

The study used a 1-group, posttest-only design to develop and evaluate immersive simulation content in nursing education.

### Procedures

**Figure 1:**
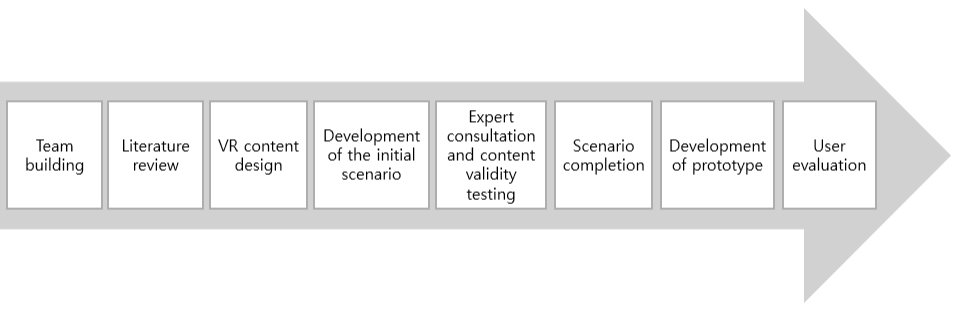
Research overview. VR: virtual reality.

The VRS content was developed via (1) team building, (2) literature review, (3) VR content design, (4) development of the initial scenario, (5) expert consultation and content validity testing, (6) scenario completion, (7) development of prototype content, and (8) user evaluation (Figure 1). This process can be broadly divided into 2 parts—development of content and user evaluation.

### Part 1: Development of Immersive Simulation Content

#### Step1: Team Building

The 5 developers (2 nursing experts, 2 IT developers, and 1 3D-modeling expert) produced the immersive content throughout the study from October 2020 to April 2021. A total of 8 nursing professionals (3 professors and 5 clinicians) supported the scenario development process.

First, the project team drafted a pediatric pneumonia nursing scenario using VR-based educational design principles and the Jeffries Simulation Theory. The 8 experts evaluated the validity of the scenario’s content.

The project team followed the design process throughout the content development and technical work. The final prototype content was iteratively refined via expert group meetings. It has been modified several times to better reflect possible clinical situations at a level that can be implemented in a virtual environment. The content validity of the developed scenario was confirmed by experts. The prototypes were categorized into 2 groups—low-fidelity prototypes using 2D materials, such as printed matter, and high-fidelity prototypes using content development programming techniques to reflect the actual environment accurately [12]. In this study, high-fidelity prototypes were developed to provide a training experience similar to that in a real clinical environment to evaluate the content applicability to nursing education.

#### Step 2: Literature Review

The project team conducted an extensive literature review to gather and analyze existing research on VR-based educational simulations. This review provided a foundation for developing the scenario and identifying best practices and potential challenges in VRS.

#### Step 3: VR Content Design

This design phase included defining the learning objectives, creating detailed storyboards for each scenario, and outlining the technical requirements for the VR environment. The design ensured that the content was pedagogically sound and technically feasible. The design phase also involved specifying the interactive elements and feedback mechanisms that would be integrated into the VR experience to enhance engagement and learning efficacy.

#### Step 4: Development of the Initial Scenario

Detailed flowcharts were created to visualize the learner’s journey through the simulation, ensuring that each step aligned with the intended learning outcomes. The initial scenario for pediatric pneumonia was developed, integrating the educational design principles and the Jeffries simulation theory. This scenario included specific nursing tasks and clinical decisions that learners would need to perform and make during the simulation.

#### Step 5: Expert Consultation and Content Validity Testing

An expert group of 8 nursing professionals with practical clinical training in pediatric nursing, including 3 pediatric nursing professors, evaluated the adequacy of the assessment data, nursing diagnoses, nursing interventions, and content relevance of the nursing evaluation algorithms. Scenario content validity testing resulted in an overall content validity index of 0.99 and 1 of 32 items was removed as “not relevant” or “somewhat relevant.” In module 4, among the assessment data to make a nursing diagnosis of skin integrity disorder, unnecessary assessment data were deleted according to expert opinion. The final version of the scenario was developed after modifications and refinements based on expert opinions.

#### Step 6: Scenario Completion

The simulation scenario included 6 modules, covering the period from admission to discharge for a child with pneumonia (Table 1). First, a disease clinical path for pneumonia was created, and based on this, 6 modules were constructed focusing on the medical treatment and nursing intervention that a 7-year-old child diagnosed with pneumonia would receive from the first day of hospitalization to the day of discharge. The scenario consisted of 6 modules following the clinical pathway of pediatric pneumonia; the nursing process was applied to each module based on detailed learning goals. The scenarios were configured to provide feedback on each learner’s performance.

**Table 1:** Virtual reality simulation modules.

Module	Day	Nursing task
Module 1	Admission	Admission assessment
Module 2	Hospital day 1-1	Fever management and medication
Module 3	Hospital day 1-2	Oxygen therapy and monitoring
Module 4	Hospital day 2	Antibiotic skin test and intravenous injection
Module 5	Hospital day 3	Respiratory therapy (nebulizer application)
Module 6	Discharge	Discharge nursing care

#### Step 7: Development of Prototype Content

The final prototype content was developed using Unreal Engine 4.25 (Epic Games) and includes 2 unique technical features. First, learners performed all procedures using their hands directly without handheld devices. Second, learners could use voice interaction with virtual agents to enhance communication competencies. To implement the VR experience, Vive Pro HMD (HTC) and a hand-tracking software development kit was used to obtain the user’s natural hand position information. Voice communication was implemented using Google’s speech kit and application programming interface.

Each module took 20-30 minutes to complete and included key nursing procedures. After completing the scenario simulation, the learner received system feedback using a visual checklist. In summary, the developed prototype provides an immersive and interactive VR experience for enhancing nursing competencies through hands-on practice and voice communication with virtual objects.

Specifically, we tested this prototype content based on feedback from the nursing expert. They indicated difficulties with voice recognition, as the system did not always accurately recognize spoken keywords. To address this, we incorporated a synonym learning algorithm using Google’s synonym crawling technology to enhance the system’s ability to understand and respond to varied inputs. This improvement aimed to provide a more seamless and intuitive user experience.

### Part 2: User Testing

**Figure 2:**
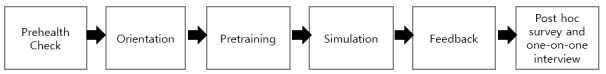
Flow of the user test.

**Figure 3:**
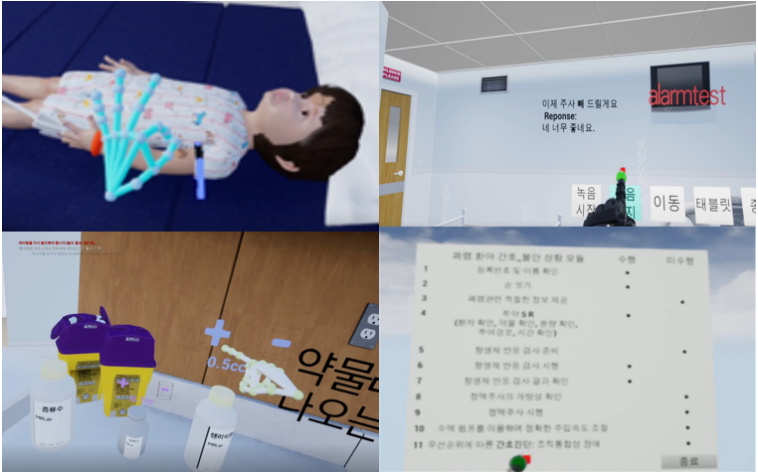
Example of screens from the developed virtual reality content.

The feasibility of this VRS content was evaluated using a survey (quantitative method) and an interview (qualitative method). Before user evaluation, the participant’s health was assessed. Immediately following the VRS experience, participants completed a survey and semistructured, in-depth, one-on-one interviews (Figure 2). Part 2 began in April 2021 (Figure 3).

### Participants

Participants were recruited by convenience sampling of nursing graduates of a university in Hanyang. Selection criteria included preservice nurses with clinical practice and simulation training experience. After obtaining approval from the institutional review board, a recruitment notice was posted in the university’s KakaoTalk chat room for nursing graduates. Those who volunteered to participate were given explanations of the study’s purpose and procedures, and all participants provided written consent. A total of 12 nursing students were selected for participation to assess the feasibility of, and satisfaction with, the developed VRS content and identify areas for improvement [13].

### Measurement

#### Overview

Participants completed an 85-item questionnaire based on previous studies; items included presence, VR technological elements, cybersickness, learning satisfaction, and participant characteristics (age, sex, general health status, experience of motion sickness, VR experience, practical experience, and satisfaction with practical training). With permission from their developers, individual survey tools were modified to suit the study characteristics and translated and back-translated by 2 bilingual translators.

#### Checklist for Participant Health Status

A total of 3 items were used to assess the participants’ current health, including 2 items from the Screening Questionnaire by Costa et al [14] and 1 item related to dizziness.

#### Postexperience Evaluation Questionnaire

##### Presence Questionnaire

The Presence Questionnaire (PQ) was developed by Witmer and Singer [15] to measure presence in a virtual environment. It consists of 5 subscales—realism, the possibility of acting, interface quality, the possibility of examination, and self-evaluation of performance. The PQ comprises 32 items, each rated on a 7-point Likert scale (1=not at all and 7=completely).

##### Virtual Reality Systems Questionnaire

The Virtual Reality Systems Questionnaire (VRSQ) is a 22-item tool developed by Norman [16] to evaluate VR games. It consists of 7 technical elements, each rated on a 9-point Likert scale. The original VRSQ was modified to suit this study and consisted of 20 items.

##### Simulator Sickness Questionnaire

The Simulator Sickness Questionnaire (SSQ) is a cybersickness scale developed by Kennedy et al [17] to measure symptoms of discomfort in VR environments using 16 items rated on a 4-point Likert scale ranging from 0 (none) to 3 (severe). The scores for each item were summed to obtain a total score indicating the overall level of discomfort experienced by individuals in the VR environment.

##### Simulation Satisfaction Questionnaire

User satisfaction was measured using a simulation satisfaction tool developed by Wotton et al [18]. It consists of 8 items rated on a 7-point Likert scale (1=not at all and 7=very much).

#### In-Depth Interviews: Core Questions

The in-depth interviews consisted of 4 open-ended questions (1-4) first used by Servotte et al [19] in their VRS study and 2 additional questions (5 and 6). The questions were (1) what was your first impression and feeling about the VRS experience? (2) What did you find enjoyable during the VRS experience? (3) What did you find uncomfortable during the VRS experience? (4) What improvements would you suggest? (5) Compared with your previous clinical practice experience, what aspects were useful? and (6) Compared with your previous clinical practice experience, what aspects were lacking?

### Data Collection

Data collection was conducted on April 15 and 16, 2021. Led by the principal researcher, a professor in the Department of Nursing, a total of 3 researchers conducted a user test of this VRS content. Of the 3 researchers, 2 were nursing majors and 1 was an engineering major. Strict COVID-19 infection control guidelines, such as fever checks and social distancing, were followed. Using the health checklist, only participants without nausea, vomiting, or physical discomfort in the 48 hours prior to the VRS experience took part.

In our study, to minimize cybersickness, the duration of wearing the equipment per person was limited to less than 1 hour at a time. Based on these internal test guidelines, each participant was allowed to experience only 1 module of 6 pediatric pneumonia contents through random assignment using a computer in advance. Participants received the scenario via email the day before the simulation. On the day of the VRS experience, participants watched an orientation video about all 6 modules and prepracticed for approximately 40 minutes. After a 20-minute break in an open space, they participated in a user test. When the simulation experience was over, participants filled out a questionnaire and had a one-on-one personal interview with the principal researcher.

### Data Analysis

Quantitative data were analyzed using SPSS Statistics software (version 25.0; IBM Corp) with descriptive statistics, such as means, SDs, and frequencies. Qualitative interview data were analyzed using thematic analysis by Braun and Clarke [20]. Interview transcripts were coded using NVivo 12.0 Pro software (Lumivero), and member checking, continuous comparison, and interrater verification processes were implemented to enhance the reliability and validity of the analyzed qualitative data. All researchers participated in the iterative analysis process.

### Ethical Considerations

This study was conducted after obtaining approval from the institutional review board of Hanyang University (HYUIRB-202103-017). To ensure anonymity and to protect participants’ personal information, all data collected were immediately encoded in compliance with the Declaration of Helsinki.

## Results

### Participant Demographics

A total of 12 licensed nurses, 3 (25%) male nurses and 9 (75%) female nurses with a mean age of 24.3 (SD 1.23) years, participated in the VRS training (Table 2). Most respondents reported good health on a regular basis (n=11, 92%) and did not experience motion sickness in their daily lives (n=9, 75%). Only 1 person subjectively assessed her health status as unhealthy due to her underlying disease, and there were no problems during the preliminary health check. One-third of participants (n=4, 33%) had experience with VR through games or other means, and all had previous clinical training with high-fidelity simulation (HFS) using mannequins and web-based simulation with vSim for Nursing. All participants had no problems checking the preliminary health checklist.

**Table 2:** General characteristics of participants (N=12).

Characteristics and categories		Values
**Sex, n (%)**		
	Female	9 (75)
	Male	3 (25)
Age (years), mean (SD)		24.3 (1.23)
**General health status, n (%)**		
	Very unhealthy	0 (0)
	Unhealthy	1 (8)
	Healthy	7 (58)
	Very healthy	4 (33)
**Experience of motion sickness when traveling in a car, n (%)**		
	Always	0 (0)
	Mostly	3 (25)
	Rarely	9 (75)
	Never	0 (0)
**VR^a^ experience such as games, n (%)**		
	No	8 (67)
	Yes	4 (33)
**Clinical practice experience, n (%)**		
	1 semester	1 (8)
	2 semesters	7 (58)
	3 or more semesters	4 (33)
**Practice experience outside of hospitals (one or both), n (%)**		
	vSim for Nursing	12 (100)
	HFS^b^	2 (17)
**Satisfaction with previous practical training, n (%)**		
	Very dissatisfied	0 (0)
	Dissatisfied	0 (0)
	Neutral	3 (25)
	Satisfied	9 (75)
	Very satisfied	0 (0)

^a^VR: virtual reality.

^b^HFS: high-fidelity simulation.

### Presence

The mean presence score during the VRS training was 4.01 (SD 1.43) out of 7 points, which is moderate. High scores were achieved for the following items: “How well could you identify sounds?” (mean 5.50, SD 1.09), “How well could you localize sounds?” (mean 5.42, SD 1.68), “How proficient in moving and interacting with the virtual environment did you feel at the end of the experience?” (mean 5.17, SD 1.53), and “To what extent did the visual aspects of the environment produce user involvement?” (mean 5.10, SD 1.20). Items with low scores were “How natural was the mechanism that controlled movement through the environment?” (mean 2.40, SD 0.90) and “How compelling was your sense of moving around inside the virtual environment?” (mean 2.50, SD 1.24; Table 3).

**Table 3:** Presence (N=12)^a^.

Item	Values, mean (SD)
How much were you able to control events?	3.30 (1.20)
How responsive was the environment to actions that you initiated (or performed)?	3.10 (0.80)
How natural did your interactions with the environment seem?	3.10 (1.10)
How completely were all of your senses engaged?	4.80 (1.40)
How much did the visual aspects of the environment involve you?	5.10 (1.20)
How much did the auditory aspects of the environment involve you?	4.00 (1.30)
How natural was the mechanism which controlled movement through the environment?	2.40 (0.90)
How aware were you of events occurring in the real world around you?	3.80 (1.60)
How aware were you of your display and control devices?	4.80 (1.50)
How compelling was your sense of objects moving through space?	3.75 (1.55)
How inconsistent or disconnected was the information coming from your various senses?	3.83 (1.03)
How much did your experiences in the virtual environment seem consistent with your real-world experiences?	3.75 (1.42)
Were you able to anticipate what would happen next in response to the actions that you performed?	4.67 (1.50)
How completely were you able to actively survey or search the environment using vision?	4.42 (1.78)
How well could you identify sounds?	5.50 (1.09)
How well could you localize sounds?	5.42 (1.68)
How well could you actively survey or search the virtual environment using touch?	3.00 (1.41)
How compelling was your sense of moving around inside the virtual environment?	2.50 (1.24)
How closely were you able to examine objects?	3.75 (1.60)
How well could you examine objects from multiple viewpoints?	4.00 (1.71)
How well could you move or manipulate objects in the virtual environment?	3.67 (0.98)
To what degree did you feel confused or disoriented at the beginning of breaks or at the end of the experimental session?	3.00 (1.54)
How involved were you in the virtual environment experience?	4.92 (1.68)
How distracting was the control mechanism?	4.17 (1.70)
How much delay did you experience between your actions and expected outcomes?	3.75 (1.96)
How quickly did you adjust to the virtual environment experience?	4.58 (1.24)
How proficient in moving and interacting with the virtual environment did you feel at the end of the experience?	5.17 (1.53)
How much did the visual display quality interfere or distract you from performing assigned tasks or required activities?	3.33 (1.23)
How much did the control devices interfere with the performance of assigned tasks or with other activities?	4.92 (1.31)
How well could you concentrate on the assigned tasks or required activities rather than on the mechanisms used to perform those tasks or activities?	2.75 (1.66)
Did you learn new techniques that enabled you to improve your performance?	3.33 (1.78)
Were you involved in the experimental task to the extent that you lost track of time?	4.67 (2.06)

^a^Total: mean 4.01 (SD 1.43).

### VR Systems

The mean score for technical elements of the VR system was 4.91 (SD 0.81) out of 9. High scores were achieved for “Auditory glitches” (mean 7.30, SD 1.70), “Trying to locate the source of sounds” (mean 6.92, SD 1.73), “Trying to turn and see what is to the left or right”(mean 6.58, SD 1.93), and “Trying to turn and see what is behind” (mean 6.42, SD 2.02), which indicates a few technical problems. Low scores were achieved for “Trying to aim or point targets with skeletal hands” (mean 3.83, SD 2.29), “Calibrating the system and tracking” (mean 3.80, SD 1.80), and “Trying to aim or point targets with robotic hands” (mean 3.42, SD 1.93).

### VR Sickness (Simulator Sickness)

The mean score for VR sickness during the training was 0.64 (SD 0.35) out of 3, indicating that most participants did not experience cybersickness. Only minimal discomfort (1 point) was reported for most items. However, scores for eye strain (mean 1.60, SD 1.20), fatigue (mean 1.40, SD 0.80), and head fullness (mean 1.0, SD 0.95) were ≥1 point, indicating some symptoms (Table 4).

**Table 4:** Virtual reality sickness (N=12)^a^.

Item	Values, mean (SD)
General discomfort	0.40 (0.70)
Fatigue	1.40 (0.80)
Headache	0.80 (1.00)
Eyestrain	1.60 (1.20)
Difficulty focusing	0.80 (0.80)
Increased salivation	0.00 (0.00)
Sweating	0.40 (0.90)
Nausea	0.30 (0.90)
Difficulty concentrating	0.40 (0.70)
Fullness of head	1.00 (0.95)
Blurred vision	0.75 (1.06)
Dizzy (eyes open)	0.92 (0.67)
Dizzy (eyes closed)	0.83 (0.94)
Vertigo	0.50 (0.67)
Stomach awareness	0.00 (0.00)
Burping	0.00 (0.00)

^a^Total: mean 0.64 (SD 0.35).

### Simulation Satisfaction

The overall mean score (8 items) for user satisfaction with the VRS training program was 5.00 (SD 1.00) out of 7. The highest scores (mean 5.30, SD 1.20) were related to experience enjoyment and problem-solving opportunities provided by the VRS training program. Items that received lower scores were those asking whether the participants could concentrate during the VRS training (mean 4.80, SD 1.40), whether the challenges were appropriate (mean 4.80, SD 1.40), and whether feedback provided during the VRS training helped them improve nursing knowledge (mean 4.80, SD 1.70; Table 5).

**Table 5:** Simulation satisfaction^a^.

Item	Values, mean (SD)
Enjoyment of the virtual reality simulation practice	5.30 (1.20)
Ability to concentrate during the virtual reality simulation	4.80 (2.00)
Provision of appropriate challenges	4.80 (1.40)
Opportunity for problem-solving	5.30 (1.20)
Usefulness in learning on hospitalized children with pneumonia	5.10 (1.20)
Usefulness in improving pediatric nursing practice skills	4.90 (1.40)
Usefulness of feedback in patient care	4.90 (1.50)
Usefulness of feedback in enhancing nursing knowledge	4.80 (1.70)

^a^Total: mean 5.00 (SD 1.00).

### Qualitative Evaluation

#### Overview

By analyzing the one-on-one in-depth interview data of the 12 participants, the strengths, weaknesses, improvement requirements, and comparison points of the content developed in this study were confirmed.

#### Strengths of VRS Content

Of the 12 participants, 9 (75%) responded that the visual features were well implemented, and they enjoyed manipulating and moving objects with their hands in the virtual environment. Some participants also found the virtual interactive experiences fascinating and were pleased to apply what they learned in practice.

I enjoyed the feeling of interaction and communication in the virtual environment as if I were observing the patient’s reactions during real practice.

Participant 7

#### Weaknesses of VRS Content

Participants reported difficulties with voice recognition because communication centered on specific keywords and with technical aspects of virtual object manipulation. Additionally, some participants experienced disruptions during the nursing procedure owing to instability in certain virtual spaces.

It was frustrating when my words were not recognized properly, resulting in a generic response such as “Yes, I understand” to whatever I said.

Participant 6

### Comparison With Existing Practical Training

#### Strengths of VRS Content

Participants noted that traditional hospital–based clinical training often involves fragmented training based on situational needs, whereas they mentioned the most significant advantage of VRS is the ability to experience independent nursing practice from beginning to end. They also found it highly beneficial to experience the entire continuum of nursing care, from admission to discharge, for specific diseases, and they highly appreciated the absence of time and space constraints in VRS.

In the actual clinical practice setting, we surround the patient like a folding screen, and there are many things we cannot see due to patient privacy. This makes us feel like we are gathering skills bit by bit rather than acquiring them systematically. However, with VR, we can perform the entire procedure from start to finish. In that sense, it is better than clinical practice.

Comparison with clinical practice: participant 11

Our group consisted of only 4 members, with some taking on the roles of nurse or doctor, while others were responsible for taking pictures or did not have the opportunity to perform nursing skills, which was disappointing. However, doing everything by myself allowed me to focus on improving my nursing skills.

Comparison with HFS: participant 4

Other programs like vSim for Nursing do not provide two-way communication. However, with this program, I felt like there was some two-way communication depending on what I said or did. It was fun and felt like I was a real nurse in the virtual environment, communicating and receiving reactions from a caregiver in my presence.

Comparison with web-based simulations: participant 10

#### Weaknesses of VRS Content

Compared with actual clinical practice, the limited manipulation of nursing supplies was evaluated as disappointing in that students were unable to perform detailed movements. Some pointed out that they had difficulty immersing themselves in the virtual clinical situation because they were concentrating on manipulating objects.

The simulations were limited to predetermined scenarios, whereas unexpected situations and various patient reactions may be encountered in a hospital setting. The tension that accompanies these situations may be lacking in simulations because all students are required to complete the same simulation from start to finish.

Comparison with clinical practice: participant 2

During the simulation, I was able to practice communication while working with my friends as a team. Unfortunately, I was doing everything alone in this program.

Comparison with HFS: participant 5

Communicating as if it were reality was helpful, but it was uncomfortable when my words were not accurately recognized.

Comparison with a simple web-based simulation: participant 4

## Discussion

### Principal Findings

VRS can provide realistic learning experiences through lifelike 3D environments, enhancing immersion and presence [21]. In this study, we sought to develop effective immersive nursing content by maximizing the advantages of VR and the learning effect in nursing through a multidisciplinary approach.

The content was developed using VR-based simulation design to create a realistic environment, considering clinical issues and contextual factors, while incorporating learning elements, such as clues and hints for learning and evaluation. Emphasis was placed on technical features, including visual implementation, to enhance immersion and presence and the educational design of the content [9]. Immersion relates to the technical aspect of VRS as an objective characteristic of the development environment, whereas presence refers to the user’s psychological and subjective perception that they are present in the immersive virtual environment [19]. Immersion can evoke a feeling of physical “presence” by providing an experience similar to real-world ones [22].

The research team held numerous discussions and participated in refinement with modeling experts to enhance immersion by increasing visual fidelity and creating a more realistic environment. Because vision is the primary sense for perceiving information, high immersion and presence are needed in educational content design [23]. Most respondents rated the visual implementation as excellent, which helped them maintain high levels of immersion. However, some participants noted that engagement was hampered in part by the unsophisticated nursing supplies and the racial inconsistency of the caregiver avatars. This mismatch partially disrupted their immersion [24]. In future VR content production, it will be essential to consider not only the color, brightness, and spatial perception of virtual objects but also learners’ cultural characteristics.

The main goal of the content development in this study was to enhance the sense of presence and provide a practice environment that would allow learners to perform nursing procedures with their own hands and provide emotional support to a virtual patient through verbal communication. According to user evaluations, multimodal interaction with voice communication and implementation of direct actions in the environment were viewed as positive and provided a moderate level of presence. However, communication accuracy and technical features related to virtual object manipulation required improvement. To the best of our knowledge, this is the first study to combine voice interactions with direct hand manipulations in practical nursing training. Multimodal interaction technology can increase resemblance to reality and provide a deeper sense of immersion, enhancing learning effectiveness [25,26]. Future studies should aim to improve the accuracy of multimodal interactions.

Despite the demand for improvement of virtual object manipulation and communication accuracy, satisfaction with the VRS program was relatively high, rated as moderate or better. The 2 most highly rated items were related to joyful experiences and problem-solving opportunities. These results align with the work by Lin et al [27] on multimodal interactions in VR for thoracic diagnostic scenarios, underscoring the effectiveness of such technologies in creating immersive educational experiences. This indicates the strong educational potential of immersive VRS. If immersive simulation content is developed by focusing on the strengths of VRS compared with existing training methods, it will be more effective in future nursing practice education.

The participants stated that the biggest advantage of VRS compared with traditional clinical practice was the opportunity to directly experience nursing procedures independently from start to finish. This finding is consistent with a previous study on multiuser virtual environment simulation experiences for nursing students [28], which highlighted building a “frame of thinking like a nurse” as a major theme. This self-directed nursing experience can facilitate learners’ metacognition and enhance competence [28,29]. The participants suggested that this immersive content could improve nursing students’ critical thinking and problem-solving skills. In VRS training, learners make clinical decisions and perform nursing activities based on their own judgments, which can effectively enhance critical thinking and problem-solving skills [5]. A previous study on nursing students [30] also confirmed the usefulness of VRS for developing clinical decision-making skills. However, this preliminary study was limited by time, and each participant experienced only 1 module. Follow-up studies are required to confirm the effectiveness of VR-based training.

Another strength of VRS pointed out by participants was the ability of students to experience the entire nursing process for patient care based on theoretical knowledge. In this study, participants were unable to experience all 6 modules due to time constraints while wearing VR, but the entire flow of all 6 modules was shown through video material during orientation on the day of the experience. Participants experienced the entire nursing process on a specific day on their own within 1 randomly selected module. This highlights the value of the full-cycle scenario design based on the clinical pathway of a corresponding illness. Traditional clinical training often limits nursing students to fragmented or random opportunities situations rather than structured, comprehensive practice sessions for a given condition [31]. Although traditional clinical training allows for an experience of unexpected situations, it lacks structure. Well-structured VRS practical training content design allows learners to apply their theoretical knowledge to practical situations systematically. Thus, VRS will be useful for reducing gaps between theoretical knowledge and practical skills.

Because VRS has some drawbacks, that is, lack of actual clinical practice and teamwork scenarios, it may be more effective to integrate VRS into traditional clinical training, allowing the two training methods to complement one another. Immersive VRS content can expand learning experiences into previously inaccessible quantitative and qualitative areas [32]. To improve the quality of practical nursing training in the post–COVID-19 era, a systematic education design model that leverages the advantages of VR and real-world training to optimize nursing education is needed.

### Limitations and Future Research

The COVID-19 pandemic posed certain challenges for this study, particularly regarding participant recruitment and experiment execution. These constraints resulted in a smaller sample size, affecting the findings’ generalizability. Additionally, the duration of the VR simulation was limited to minimize the cybersickness risk, and each participant experienced only 1 module instead of the entire hospitalization scenario.

Despite these limitations, the study significantly contributes by systematically developing immersive VRS educational content for nursing. Using the Jeffries Simulation Theory template and VRS education design principles, the study confirmed the feasibility of this content in practical nursing training. Importantly, the study implemented multimodal interaction, enabling learners to use their hands directly and engage in voice communication, thereby closely simulating real clinical nursing practices.

Future research should address these limitations by recruiting a larger and more diverse sample population. Allowing participants to experience full-cycle clinical pathways of diseases will facilitate a more comprehensive verification of learning effects, enhancing the validity and applicability of the results. Furthermore, expanding the scope of multimodal interactions and refining the VR environment will improve the realism and effectiveness of the education, establishing it as an even more robust tool for nursing education.

### Conclusions

We developed multidisciplinary VRS educational content that integrates the characteristics of nursing, education, and engineering and confirmed its feasibility as effective simulation education. Compared with conventional practical nursing training, this VRS content was valuable, allowing students to perform tasks independently and to experience the overall flow of nursing. The physical training environment was well-implemented visually, and the multimodal interaction increased immersion and presence. The VRS was also useful for enhancing individual nursing competencies through one-on-one training. However, keyword-based voice interactions were a major obstacle to immersion, highlighting the need for additional research. Future research should explore the detailed learning effects and educational possibilities of immersive content using multidisciplinary design models. In conjunction with conventional clinical practice and HFS, VRS content based on a multidisciplinary educational model can be used in practical nursing training in the post–pandemic era to optimize clinical competency.

## Abbreviations

HFS: high-fidelity simulation
NLN: National League for Nursing
PQ: Presence Questionnaire
SSQ: Simulator Sickness Questionnaire
VR: virtual reality
VRS: virtual reality simulation
VRSQ: Virtual Reality Systems Questionnaire
